# ﻿Checklist of Orchidaceae from Caquetá, Colombia

**DOI:** 10.3897/phytokeys.229.102737

**Published:** 2023-07-06

**Authors:** Tatiana Arias, Jeisson Chaux-Varela, Maria del Pilar Camero, R. Alexis Calderon-Álvarez, Anyi Carolina Trujillo, Marco A. Correa-Munera, Alejandro Zuluaga, Oscar Perdomo, Oscar A. Pérez-Escobar, Edwin Trujillo-Trujillo, Janice Valencia-D.

**Affiliations:** 1 Marie Selby Botanical Gardens, Sarasota FL, USA; 2 Sociedad Colombiana de Orquideología, Medellín, Antioquia, Colombia; 3 Departamento de Biología, Universidad de la Amazonia, Florencia, Caquetá, Colombia; 4 Grupo de Investigación en Ecología y Territorio, Departamento de Ecología y Territorio, Pontificia Universidad Javeriana, Bogotá D.C., Colombia; 5 Jardín Botanico Uniamazonia and Herbario Enrique Forero – HUAZ, Florencia, Caquetá, Colombia; 6 Departamento de Biología, Universidad del Valle, Cali, Valle del Cauca, Colombia; 7 Grupo de Investigación en Agroecosistemas y Conservación en Bosques Amazónicos GAIA, Universidad de la Amazonia, Florencia, Caquetá, Colombia; 8 Royal Botanic Gardens, Kew, Richmond, UK; 9 Laboratorio de Agrobiodiversidad y Malherbologia LAMUA, Universidad de la Amazonia, Florencia, Caquetá, Colombia

**Keywords:** Alpha diversity, Amazon, Andes, floristic studies, foothills, orchids

## Abstract

A checklist of Orchidaceae from Caquetá, Colombia is presented here. We recorded 98 genera and 418 species, exceeding a previous inventory by 276 species. The checklist is conservative in the number of genera and species by including only taxa that were fully and reliably identified and that are either linked to a corresponding herbarium voucher, a living collection specimen or a photo taken in the field and published in iNaturalist by one of the authors or a collaborator. The documented species diversity in the region could dramatically increase in the next few years with additional collecting efforts in the eastern slopes of the Andes nested in Caquetá. About 9% (418/4600) of all Orchidaceae species recorded for Colombia are reported for this area, showing the important contribution to orchid diversity of Andean-Amazonian foothills of Caquetá.

## ﻿Introduction

Orchidaceae are one of the most diverse and widely distributed flowering plant families including 25,000–27,000 species and 880 genera (Chase et al. 2015). Colombia has the largest diversity of orchid species in the American tropics (Pérez-Escobar et al. 2022a), hosting ~ 4,600 species that represent ~18% of the known species diversity in the family. The highest level of species richness arises in the northern Andes region of the country (Pérez-Escobar et al. 2022a), where a large number of endemic species occur, accounting for 36.8% of the total species reported for Colombia. With new orchid novelties published annually (Ortiz Valdivieso et al. 2009; Hágsater et al. 2013; Pérez-Escobar et al. 2021, 2022b; Vieira-Uribe and Moreno 2022), Colombia is a hotspot for biodiversity conservation (Betancur et al. 2015).

**Figure 1.**
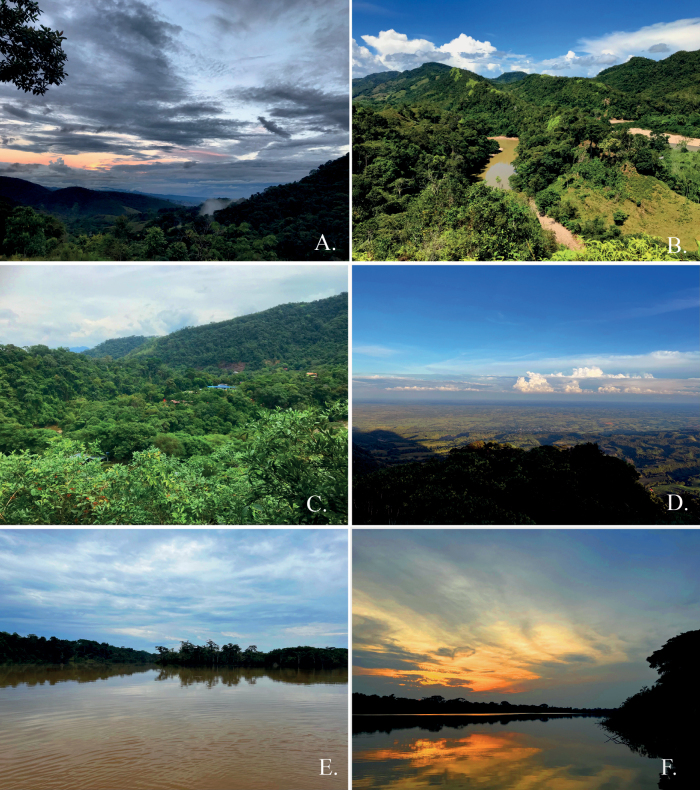
Representative landscapes from Caquetá, Colombia. **A** general view of the Andean Piedmont in the Municipality of Florencia **B** Hills and rivers coming down from the eastern slopes of the Andes and flowing into the Amazonian Forest in the Municipality of Belen de los Andaquíes, the Pescado River **C** Hilly slopes from the Andean Piedmont transitioning to the Amazon Forest in the Municipality of Belen de los Andaquíes **D** General view of Amazonia Forest from a hilly slope of the eastern Andes, in the Municipality of Paujíl **E, F** Amazonian waterlogged Forest at La Laguna de Peregrinos, Municipality of Solano.

Caquetá, one of Colombia’s 32 Departments, is a largely unexplored region with an extraordinary ecosystem diversity, geographically presenting a variety of landscapes, topographic forms and different types of associated vegetation and water sources, including the Amazon plains, valleys, hills, foothills and mountain ranges (Fig. 1). The Department contains four national natural parks, covers part of the Chiribiquete World Heritage Park and 35 recognised civil society reserves (RUNAP 2022, webpage checked June 2023). Caquetá is placed in the eastern slopes of the Andean foothills, a confluence zone of mountainous and lowland Amazonian landscapes with different communities’ composition. The Andean foothills of Caquetá range between 200 and 1000 m a.s.l.

The Colombian Eastern Andean Mountain range transitions along an environmental gradient from foothills to either the Guyana Shield (Meta and Caquetá), the Amazon Basin (Caquetá, Putumayo and Amazonas) or the Orinoco Basin (Arauca, Casanare and Meta) (Hoorn et al. 2010). These ecotones are hyperdiverse because of the evolutionary, biogeographical and ecological processes that operate in a rich array of landscapes (Ruiz et al. 2007; Instituto Geográfico Agustín Codazzi 2010). It is perhaps in the confluence of lowland and mountainous landscapes where the greatest wealth of plant species diversity and endemism occurs in the country (Ruiz et al. 2007; Pérez-Escobar et al. 2022a), but the limited existing orchid inventories underestimate the region’s species diversity.

Although Orchidaceae are diverse within Caquetá, few checklists and taxonomic studies focusing on this group are available. For example, the Catalogue of Plants and Lichens of Colombia (Bernal et al. 2016), reported a total of 104 orchid species, whereas “The National Orchid Conservation Plan” presented a count of 142 species (Betancur et al. 2015). Currently, Caquetá is severely affected by deforestation driven by anthropogenic transformations of the natural ecosystems (Jaramillo-Castelblanco 2016; IDEAM 2020). Biological diversity inventories of the Andean-Amazonian Region are, thus, crucial to provide information for habitat conservation strategies in the region.

In this study, we generated a detailed species list of Orchidaceae for the Department of Caquetá, one of the most unexplored areas in Colombia, due to, amongst other factors, difficulties such as security risks and lack of easy access routes to some of its regions and municipalities. This is a collective work developed by more than twelve Colombian botanists during 2019–2023, under the umbrella project “Orquídeas para la Paz” (Orchids for Peace). This programme aims to explore, reproduce and support orchid species recovery, while developing sustainable strategies for business, based on horticulture for vulnerable communities around Colombia. Data obtained here come from living collections, photographs taken from field and an extensive review of herbarium collections around the world.

## ﻿Methods

### ﻿Study area

Caquetá is located in south-western Colombia, between latitudes 0.7°S–2.9°N and longitudes 71°–76°W. It comprises 16 municipalities including Florencia, Belén de los Andaquíes, El Paujil, Doncello and La Montañita, amongst others (Table 1). This region contains a variety of landscapes and ecosystems ranging from the Eastern Cordillera of the Andes to the Amazonian plains, with elevations ranging from 0–3200 m a.s.l. The mean annual rainfall is about 2179 mm. The mean annual temperature ranges from 27–29 °C (Instituto Geográfico Agustín Codazzi 2010) (Fig. 1).

**Table 1.** Checklist of the Orchidaceae of Caquetá, Colombia. ALB: Albania, BEL: Belen de los Andaquies, CAR: Cartagena el Chaira, CUR: Curillo, DON: Doncello, FLO: Florencia, MIL: Milan, MOR: Morelia, PAU: Paujil, PRC: Puerto Rico, SJF: San Jose del Fragua, SOL: Solano, STA: Solita, SVC: San Vicente del Caguán, VAL: Valparaiso. * Endemic to Colombia ** New report for Colombia.

Species name	Accessions reviewed	Area
*Aciantheracasapensis* (Lindl.) Pridgeon & M.W. Chase	iNaturalist	BEL
*Aciantheraciliata* (Knowles & Westc.) F. Barros & L. R. S. Guim.	*Pabon M. 320* (COAH), *Arias T. 851, 852, 914* (HUAZ)	SOL, VAL
*Aciantheradiscophylla* (Luer & Carnevali) Luer	Living collection (El Manantial, Florencia)	SOL
*Aciantheraerinacea* (Rchb. f.) A. Doucette	*Arias T. 951, Chaux-Varela J. 61* (HUAZ)	FLO, PAU, SVC
*Aciantherasicaria* (Lindl.) Pridgeon & M. W. Chase	*Arias T. 917* (HUAZ)	FLO
*Aganisiacyanea* (Lindl.) Rchb.f.	*Arevalo R. 406* (COAH), *Vasco A. 389, 396, 360, 420* (HUA), *Trujillo E. 1043* (HUAZ)	BEL, SOL
*Aganisiafimbriata* Rchb.f.	*Torres M.M. 1084* (COAH), *Mesa N. & Trujillo E. 24* (HUAZ)	FLO, SOL
*Anathallisacuminata* (Kunth) Pridgeon & M. W. Chase	*Benavides A. 506* (HUA), *Arias T. 969* (HUAZ)	DON, SOL
*Anathallisbrevipes* (H. Focke) Pridgeon & M. W. Chase	*Arias T. 906* (HUAZ)	CAR
*Anathallisspiculifera* (Lindl.) Luer	*Sanchez M. 1834, 1835* (COAH)	SOL
*Anathallissclerophylla* (Lindl.) Pridgeon & M. W. Chase	iNaturalist	SVC
*Aspidogyneclavijera* (Rchb. f.) Meneguzzo	*Cardenas D. 45472* (COAH)	MOR
*Aspidogyneconfusa* (C. Schweinf.) Garay	*Castro F. 9890* (COAH)	SOL
*Aspidogynefoliosa* (Poepp. & Endl.) Garay	*Trujillo E. 7026, 7109* (CUVC)	ALB
*Aspidogynejamesonii* (Garay) Meneguzzo	*Romero 4050* (COL)	CAR
*Batemanniacolleyi* Lindl.	*Arevalo R. 43* (COL), *Vasco A. 158* (HUA)	FLO, SOL
*Beloglottiscostaricensi*s Schltr.	iNaturalist	FLO
*Bifrenariaclavigera* Rchb.f.	*Trujillo E.* 1043 (COAH)	BEL
*Bifrenarialongicornis* Lindl.	*Arbelaez E.* 907 (COAH), *Arevalo R. 417* (COL), *Vasco A. 264* (HUA)	SOL
*Braemiavittata* (Lindl.) Jenny	*Duivenvoorden J. 2362*, *Torres M. 1128* (COAH), *Benavides A. 634* (HUA)	SOL
*Brachionidiumlehmannii* Luer **	*Arias T. 952* (HUAZ)	PAU
*Brassiacaudata* (L.) Lindl.	*Ortiz-Valdivieso M. 596* (HPUJ), *Arias T. 857* (HUAZ)	BEL
*Bulbophyllumlehmannianum* Kraenzl.	*Arevalo R. 363* (COL)	FLO
*Campylocentrumkuntzei* Cogn. ex Kuntze	*Correa M. 7136* (COAH, HUAZ)	FLO
*Campylocentrummicranthum* (Lindl.) Maury	iNaturalist	SVC
*Catasetumdiscolor* (Lindl.) Lindl.	*Arbelaez E. 238* (HUA), *Idobro J. M. 9001* (COL), *Arevalo R. 311* (COL), *Sastre R. D. 5173* (P)	SOL
*Catasetumochraceum* Lindl.	*Gonzalez M. F. 2680, 2710* (COL)	SOL
*Catasetumroseo-album* (Hook.) Lindl.	*Barbosa C.* 7716 (FMB)	SOL
*Catasetumtabulare* Lindl.	*Perdomo O.* 430 (CUVC)	FLO
*Catasetumtuberculatum* Dodson C.	*Aguilar M. 253* (COAH), *Arias T. 858* (HUAZ)	FLO
*Catasetumvillegasii* G. F. Carr	*Carr G. F.* (COAH, USF)	SVC
*Cattleyacrispa* Lindl. **	Living collection (El Manantial, Florencia)	CAR, SJF
*Cattleyaviolacea* (Kunth) Rolfe	*Trujillo E. 808, Calderon A. A. 263* (HUAZ)	CAR
*Chondrorhyncharosea* Lindl.	*Schmidt-Mumm K. s.n.* (LA)	FLO
*Cleistesabdita* G. A. Romero & Carnevali	*Palacios P. 582, Castaño N. 3184* (COAH)	FLO, SOL
*Cleistesrosea* Lindl.	*Jaimes M. S. 1269* (COAH), *Sanin D. 6454* (COL), *Perdomo O. 256* (CUVC), *Cumaco L. S. & Trujillo E. 42, Arias T. 990, Chaux-Varela J. 96* (HUAZ)	FLO, PAU
*Cleistestenuis* (Griseb.) Schltr.	*Aguilar M. 253* (COAH)	SOL
*Coryanthesleucocorys* Rolfe	iNaturalist	BEL, FLO
*Cranichispolyantha* Schltr.	*Madero 22* (AMES)	N/A
*Cryptarrhenalunata* R. Br.	iNaturalist	FLO
*Cycnochesegertonianum* Bateman	*Perdomo O. 387* (CUVC)	FLO
*Cycnocheshaagii* Barb. Rodr.	*Dodson C. 3249* (COAH)	DON
*Cyrtochilumcaquetanum* P. Ortiz-Valdivieso M, L. E. Álvarez & A. J. Carrillo	*Ortiz-Valdivieso M. 1393* (HPUJ)	N/A
*Cyrtochilumcimiciferum* (Rchb. f.) Dalström	iNaturalist	SVC
*Cyrtochilumdivaricatum* (Lindl.) Dalström	iNaturalist	SVC
*Cyrtochilumflexuosum* Kunth	*Ramirez J. G. 5282* (JAUM), *Gentry A. et al. 9046* (MO)	FLO
*Cyrtochilummeirax* (Rchb. f.) Dalström	*Perdomo O. 416, 404* (CUVC), *Arias T. 925, 954, Chaux-Varela J. 58, 60, 92* (HUAZ)	FLO, PAU, PRC
*Cyrtochilummidas* Dalström	*Perdomo O. 0195* (CUVC)	FLO
*Cyrtochilumorgyale* Kraenzl.	iNaturalist	SVC
*Cyrtochilumporrigens* (Rchb. f.) Kraenzl.	*Perdomo O. 394, 400* (CUVC), *Calderon A. 250, 251* (HUAZ)	PRC, FLO
*Cyrtochilumramosissimum* (Lindl.) Dalström	*Trujillo E. 7587* (CUVC)	PRC
*Cyrtochilumscabiosum* Kraenzl.	*Cuatrecasas J. 8466* (COL)	FLO
*Cyrtochilumtrifurcatum* Kraenzl.	*Perdomo O. 412* (CUVC)	PRC
*Cyrtochilumundulatum* Kunth	iNaturalist	FLO
*Cyrtochilumventilabrum* Kraenzl.	*Perdomo O. 393* (CUVC)	PRC
*Cyrtopodiumcristatum* Lindl.	*Betancur J. 1548* (HUA)	SVC
*Cyrtopodiumpalmifrons* Rchb. f. & Warm.	Living collection (El Caraño, Florencia)	FLO
*Dichaeaancoraelabia* C. Schweinf	*Perdomo O. 267* (CUVC), *Ortiz-Valdivieso M. 531* (HPUJ), *Mesa N. & Trujillo E. 03 Arias T. 877* (HUAZ)	BEL, FLO, SOL
*Dichaeacaquetana* Schltr. *	*Fernández-Pérez A.* 7240 (COL)	FLO
*Dichaeahystricina* Rchb.f.	*Castaño N.* 8705 (COAH)	BEL, FLO
*Dichaeapanamensis* Lindl.	*Dueñas H. 3060* (COL), *Vasco A. 387* (HUA)	SOL
*Dichaeapendula* (Aubl.) Cogn.	*Castaño N. 8678* (COAH), *Betancur J. 1916* (COL, HUA)	BEL, SVC
*Dichaeapicta* Rchb.f.	*Jimenez E. 11* (COAH)	FLO
*Dichaearendlei* Gleason	*Franco-Rosselli P. 3825* (COL), *Vasco A. 220, 267, 304* (HUA), *Betancur J. 13560* (COAH)	SOL
*Dichaeasodiroi* Schltr.	*Ortiz-Valdivieso M. 553* (HPUJ)	FLO
*Dichaeasplitgerberi* Rchb.f.	*Trujillo E. 956* (COAH, FMB), *Castaño N. 1761, Cardenas D. 40460, 44494, 48527* (COAH)	BEL, PRC, SVC, VAL
*Dichaeatrinitensis* Gleason **	*Arias T. 885* (HUAZ)	SOL
*Dichaeatrulla* Rchb.f.	*Cardenas D. 42199* (COAH), *Betancur J. 20668, Arevalo R. 98, 306* (COL), *Vasco A. 305* (HUA), *Perez* 663 (FMB)	BEL, FLO, SOL
*Dimerandraemarginata* (G. Mey.) Hoehne	*Arias T. 859* (HUAZ)	FLO
*Draculaalcithoe* Luer & R. Escobar	iNaturalist	BEL
*Duckeellaadolphii* Porto & Brade	*Pabon M. 461, 462*, *Echeverry R. 3297, Palacios P. 691, 537, 1218, Duivenvoorden J. 263* (COAH), *Arbelaez E. 64* (HUA)	SOL
*Duckeellacaquetana* Szlach. & Kolan. *	*Arbelaez M. V. 64* (COAH, UGDA)	SOL
*Duckeellafernandezii* Szlach., Kolan. & Baranow *	*Fernandez 20065* (COL, UGDA)	SOL
*Elleanthusamethystinoides* Garay	*Cardenas D. 20257* (COAH)	BEL
*Elleanthusampliflorus* Schltr.	*Arias T. 1201* (HUAZ)	FLO
*Elleanthusaurantiacus* Rchb.f.	*Castaño N. 7442, 8608, Cardenas D. 46146* (COAH), *Mason H. L. 13954, Gentry A. 9036* (COL), *Arias T. 1211* (HUAZ)	BEL, FLO. SOL
*Elleanthusblatteus* Garay	*Arias T. 980, 1193, 1217* (HUAZ)	DON, FLO
*Elleanthuscolumnaris* Rchb.f.	*Fonnegra R. 5465* (HUA, MO)	FLO
*Elleanthusconifer* (Rchb.f. & Warsz.) Rchb.f.	*Jimenez E. 26* (COAH)	FLO
*Elleanthusemberanus* (Szlach. & Kolan.) J. M. H. Shaw *	*Trujillo W. et al. 968* (COAH)	BEL
*Elleanthusfractiflexus* Schltr.	*Castaño N. 8720, Betancur J. 20388, 20544* (COAH), *Arias T. 1188* (HUAZ)	BEL, FLO
*Elleanthusgraminifolius* (Barb.Rodr.) Lojtnant	*Cardenas D. 20682, Perdomo O. 318, Betancur J. 20682* (COAH) *Perdomo O. 318* (CUVC)	BEL, FLO
*Elleanthuskermesinus* Rchb. f.	*Cuatrecasas J. 8766* (COL)	FLO
*Elleanthuslancifolius* C. Presl.	*Ortiz-Valdivieso M. 459* (HPUJ), *Araujo E. & Trujillo E. 28* (HUAZ)	BEL, FLO
*Elleanthusoliganthus* Rchb.f.	*Vargas V. A. 99* (COAH), *Cardenas D. 42099* (FMB), *Cumaco L. S. & Trujillo E. 34, Santofimio L. M. & E. Trujillo E. 04* (HUAZ)	BEL, FLO
*Elleanthusrobustus* Rchb. f.	*Arias T. 1190* (HUAZ)	FLO
*Elleanthustillandsioides* Barringer	*Trujillo W. 968* (FMB), *Cardenas D. 41786, 41817* (COAH)	BEL
*Encycliaaspera* Schltr.	*Arevalo R. 210* (COL), *211* (COAH)	SOL
*Encycliachloroleuca* Neumann	Living collection (El Caraño, Florencia)	FLO
*Encyclialeucantha* Schltr.	*Pabon M. 927, Arbelaez E. 787* (COAH)	SOL
*Encycliapilosa* (C. Schweinf.) Carnevali & I. Rámirez	*Arbelaez E. 389* (COAH)	SOL
*Epidendrumacuminatum* Ruiz & Pav.	*Ortiz-Valdivieso M. 4171* (HPUJ)	FLO
*Epidendrumacutilobum* Hágsater E. & Uribe Veléz *	*Kapuler & Hascall 168* (COL), *Arias T. 979* (HUAZ)	FLO, DON
*Epidendrumamazonicoriifolium* Hágsater E.	*Cardenas D. 40246* (COAH)	FLO
*Epidendrumangulatum* Hágsater E. & J. Duarte *	*Moreno s.n.* (AMO)	FLO
*Epidendrumangustatum* (T. Hashim.) Dodson C.	*Ortiz-Valdivieso M. 461* (HPUJ)	FLO
*Epidendrumarachnoglossum* Rchb. f. ex André	*Arbelaez E. 53* (HUA)	SOL
*Epidendrumarevaloi* (Schltr.) Hágsater E.	*Hágsater E. s.n. (AMO), Ortiz-Valdivieso M. 473* (HPUJ), *Arias T. 1196* (HUAZ)	FLO
*Epidendrumarmeniacum* Lindl. **	*Perdomo O. 302* (CUVC)	FLO
*Epidendrumaura-usecheae* Hágsater, Rinc. -Useche & O. Pérez	*Arias T. 1014* (HUAZ)	SVC
*Epidendrumbarbeyanum* Kraenzl.	*Ortiz-Valdivieso M. 462* (HPUJ)	FLO
*Epidendrumborealistachyum* Hágsater E., E. Santiago & C. Fernandez	*Sanin D. 6361* (COAH), *6100* (COL), *6558, 6632* (HUA), *Correa M. & Aldana J. 7196, Mesa N. & Trujillo E. 16 Arias T. 1199* (HUAZ)	FLO
*Epidendrumbrachyrepens* Hágsater E. **	*Betancur J. 2225* (HUA)	SVC
*Epidendrumcaesaris* Hágsater E. & E. Santiago	*Estrada J. 668* (COL)	SOL
*Epidendrumcalanthum* Rchb.f. Warsz.	*Barbosa C. 8140* (COL)	SOL
*Epidendrumcalyptrandium* Hágsater E., H. Medina & Huamantupa	*Cardenas D. 41772* (COAH, FMB)	BEL
*Epidendrum* c*horandrochilum* F. Lehm. & Kraenzl.	*Perdomo O. 235* (CUVC)	FLO
*Epidendrumcleistocoleu*m Hágsater E. & E. Santiago	iNaturalist	SVC
*Epidendrumcochlidium* Lindl.	*Jimenez E. 1* (HUA)	FLO
*Epidendrumcompressibulbum* D. E. Benn. & Christenson **	*Arias T. 926* (HUAZ)	FLO
*Epidendrumcompressum* Griseb.	*Cuatrecasas J. 27127* (COL), *Trujillo W. 816* (HUA), *Mesa N. & Trujillo E. 18* (HUAZ)	CAR, FLO
Epidendrum×communis Hágsater Ined	*Arias T. 997; 998* (HUAZ)	SVC
*Epidendrumcoronatum* Ruiz & Pav	*Arias T. 863* (HUAZ)	CAR, SJF
*Epidendrumcuneatum* Schltr.	*Arias T. 960* (HUAZ)	CAR, SJF
*Epidendrumcupreum* F. Lehm. & Kraenzl.	*Ortiz-Valdivieso M. 573* (HPUJ)	FLO
*Epidendrumcylindraceum* Lindl.	*Arias T. 1204* (HUAZ)	FLO
*Epidendrumerosum* Ames & C. Schweinf.	*Hoyos s.n.* (AMO)	FLO
*Epidendrumexcisum* Lindl.	iNaturalist	SVC
*Epidendrumflexuosum* G. Mey.	iNaturalist	SVC
*Epidendrumfilamentosum* Kraenzl.	*Perdomo O. 272, 413* (CUVC) *Arias T. 988*, *Chaux-Varela J. 34, 98* (HUAZ)	FLO, DON
*Epidendrumfimbriatum* Kunth	*Arias, T. 1198, Calderon A. 256* (HUAZ)	FLO
*Epidendrumgeminiflorum* Kunth	iNaturalist	SVC
*Epidendrumibaguense* Kunth	*Cardiel J. M. 59* (COL), *Arias T. 993* (HUAZ)	DON, FLO, SOL
*Epidendrumlacustre* Lindl.	*Forero E. 9816, Palacios P. 859, 1192, 1201, 1226, Arbelaez E. 53, 731, Duivenvoorden J. 607, Sanin D. 6362* (COAH), *Restrepo D. 344* (HUA)	FLO, SOL
*Epidendrumlongicolle* Lindl.	*Cumaco L. S. & E. Trujillo E. 16* (HUAZ)	FLO
*Epidendrummacrocarpum* Rich.	*Marin C. 2896* (COAH, COL), Velayos M. 6455 (COL)	FLO, MOR, SOL
*Epidendrummacrum* Dressler	*Ortiz-Valdivieso M 528* (HPUJ, AMO)	FLO
*Epidendrummagnicallosum* C. Schweinf.	*Arevalo R. 46, 91, 165* (COL)	SOL
*Epidendrummamapachae* Hágsater, F.O. Espinosa & E. Santiago *	iNaturalist	SVC
*Epidendrummelinanthum* Schltr.	*Plazas L. L. et al. 42* (HUAZ)	FLO
*Epidendrummicrocapitellatum* Hágsater, Medina Tr. & E. Santiago *	iNaturalist	FLO
*Epidendrummicronocturnum* Carnevali & G.A. Romero	*Arevalo R. 40* (COL)	SOL
*Epidendrummicrophyllum* Lindl.	*Arevalo R. 201* (COL)	SOL
*Epidendrummora-retanae* Hágsater E.	Living collection (El Caraño, Florencia)	FLO
*Epidendrummyrmecophorum* Barb. Rodr.	*Perdomo O. 275, 284* (CUVC), *Arbelaez E. 256, 379* (HUA)	CAR, FLO, MOR, SOL
*Epidendrumnocturnum* Jacq.	*Arbelaez E. 888, Franco-Rosselli P. 3730, Sanchez M. 1943, 1942, 1941* (COAH), *Cardenas D. et al. 42156* (FMB), *Arias T. 874–876, 959, Trujillo E. 815* (HUAZ)	BEL, CAR, DON, FLO, SJF, SOL
*Epidendrumorbiculatum* C. Schweinf.	*Ortiz-Valdivieso M. 573* (HPUJ)	SVC
*Epidendrumorchidiflorum* Salzm. ex Lindl.	*Arbelaez E. 730, 815, 776, Cardenas D. 45063, 48634, Sanin D. 6642* (COAH), *Davidse G. 5612, Hermann F. J. 11257, Manson H. L. 13949* (COL), *Arbelaez E. 379* (HUA), *Araujo D. & Trujillo E. 17, Arias T. 865, Croat T. et al. 100480* (HUAZ)	CAR, FLO, SOL
*Epidendrumporphyreonocturnum* Hágsater & R. Jiménez	*Perdomo O. 179* (CAUP)	FLO
*Epidendrumportokalium* Hágsater E. & Dodson C.	*Cuatrecasas J. 9002* (COL)	FLO
*Epidendrumputumayoense* Hágsater E. & L. Sanchéz	*Valencia E. & Hágsater E. 11640* (AMO)	N/A
*Epidendrumradicans* Pav. ex Lindl.	*Polania O. L. & Trujillo E. 5* (HUAZ)	FLO
*Epidendrumrhodochilum* (Schltr.) Hágsater E. & Dodson C.	*Trujillo W. s. n.* (AMO)	BEL
*Epidendrumrhombochilum* L.O. Williams	*Betancur J. et al. 20224* (COAH)	BEL
*Epidendrumrigidum* Jacq.	*Ortiz-Valdivieso M. 471* (HPUJ), *Polania O. L. & Trujillo E. 1, Cumaco L. S. & Trujillo E. 15* (HUAZ), *Groenendijk J. s.n.* (MA)	FLO, SOL
*Epidendrumrugulosum* Schltr.	*Sanin D. 6640* (HUA)	FLO
*Epidendrumsanctae-rosae* Hágsater E., Sauleda, Uribe Vélez & E. Santiago	*Perdomo O. 322, 424* (CUVC)	FLO
*Epidendrumsaxatile* Lindl.	*Trujillo E. et al. 1038* (COAH), *Arias T. 967* (HUAZ)	BEL, DON
*Epidendrumsculptum* Rchb.f.	*Cardenas D. 41772* (COAH, FMB)	BEL
*Epidendrumsecundum* Jacq.	*Betancur J. 20457* (COAH), *Correa M. et al. 4605* (HUAZ), *Barbosa C. et al. s.n.* (MA)	BEL, FLO, PRC, SOL
*Epidendrumspilotum* Garay & Dunst.	*Escobar R. 5270* (AMO)	N/A
*Epidendrumstenobractistachyum* Hágsater E. & E. Santiago	*Cuatrecasas J. 8426* (COL)	FLO
*Epidendrumstrobiliferum* Rchb.f.	*Cardenas D. 46422* (COAH)	SOL
*Epidendrumteuscherianum* A. D. Hawkes **	*Chaux-Varela J. 102, 103, 108* (HUAZ)	DON
*Epidendrumtridens* Poepp. & Endl.	*Franco-Rosselli P. et al. 3730* (COL)	SOL
*Epidendrumtumuc-humaciense* (Veyret) Carnevali & G. A. Romero	*Arbelaez E. & Castro F. 888, Barbosa C. et al. 8154, Castro Viejo F. et al. 335, Franco-Rosselli P.* et al. *3636* (COL)	SOL
*Epidendrumuleinanodes* Hágsater E. **	*Groenendijk 33* (COAH)	SOL
*Epidendrumvinosum* Schltr. **	iNaturalist	PAU
*Epidendrumwhittenii* Hágsater E. & Dodson C.	*Coca et al. 9207b* (FAUC)	SJF
*Epistephiumhernandii* Garay	*Arbelaez E. 276* (COAH)	SOL
*Epistephiumparviflorum* Lindl.	*Castro F. 10974, Palacios P. 864* (COAH)	SOL
*Epistephiumsubrepens* Hoehne	*Duivenvoorden J. 215, Gentry A. 65171, Ospina H. 1141, Palacios P. 581, 760, 2437, Restrepo D. 9* (COAH)	SOL
*Eriopsisbiloba* Lindl.	*Arbelaez E. 172* (COAH), *Palacios P. 2415* (COL), *Barbosa C. 7631* (FMB)	SOL
*Eriopsissceptrum* Rchb.f. & Warsz.	*Cardenas D. 46424* (COAH)	SOL
*Erycinaglossomystax* (Rchb.f.) N. H. Williams & M. W. Chase	*Arias T. 961* (HUAZ)	DON
*Erycinapumilio* (Rchb. f.) N. H. Williams & M. W. Chase	*Atwood J. T. & Mora D. s.n.*	N/A
*Erycinapusilla* (L.) N. H. Williams & M. W. Chase	*von Sneidern K. 1075, Castroviejo S. 322, Perez-Arvelaez E. 370* (COL), *Betancur J. 2349* (HUA)	BEL, FLO, SVC
*Eulophiaalta* (L.) Fawc. & Rendle	*Perdomo O. 342* (CUVC), *Betancur J. 2214* (HUA)	BEL, SVC
*Galeandramacroplectra* G. A. Romero & Warford	*Galeano G. 2249* (COL)	SOL
*Galeandrastangeana* Rchb. f.	*Franco-Rosselli P. 3860* (COL)	SOL
*Galeottianegrensis* Schltr.	*Mendoza H. 10283, Cardenas D. 48408* (COAH)	SOL, SVC
*Gongoraatropurpurea* Hook.	iNaturalist	SVC
*Gongoraportentosa* Linden & Rchb. f.	Living collection (El Caraño, Florencia)	FLO
*Habenariamesodactyla* Griseb.	*Franco-Rosselli P. 2410* (COL)	SOL
*Habenariamonorhiza* Rchb. f.	*Orozco C. I. 2768* (COL), *Goudot J. s.n.* (P), *Arias T. 756, 992, 1011, Chaux-Varela J. 107* (HUAZ)	FLO, DON, PAU, PRC, SOL, SVC
*Habenariapratensis* Rchb. f.	iNaturalist	CAR
*Houlletialowiana* Rchb. f.	*Cardenas D. 46013* (COAH)	BEL
*Houlletiasanderi* Rolfe	*Perdomo O.* 307 (CUVC)	FLO
*Hylaeorchispetiolaris* (Schltr.) Carnevali & G. A. Romero	iNaturalist	BEL
*Ionopsissatyrioides* (Sw.) Rchb.f.	*Perdomo O. 345* (CUVC)	ALB, BEL
*Ionopsisutricularioides* (Sw.) Lindl.	*Trujillo E. 846* (COAH), *Idobro J. M. 8590* (COL)	CAR
*Jacquiniellaglobosa* (Jacq.) Schltr.	Living collection (El Caraño, Florencia)	FLO
*Jacquiniellateretifolia* (Sw.) Britton & P. Wilson	*Betancur J. 20543, 20658* (COAH)	BEL, FLO
*Koellensteiniagraminea* Rchb.f.	*iNaturalist*	SVC
*Laeliarosea* (Lindl.) C.Schweinf.	*Calderon A. 260, 261, Chaux-Varela J. 43* (HUAZ)	CAR, FLO
*Lepanthesagglutinata* Luer	*Cardenas D. 41869* (COAH, FMB, MO, NYCB), *Trujillo E. 7691, 7705, 7949* (CUVC)	BEL, FLO, PRC
*Lepanthesauriculata* Luer	*Trujillo E. 7693* (CUVC)	PRC
*Lepanthesdelhierroi* Luer & Hirtz **	*Arias T. 1186* (HUAZ)	FLO
*Lepanthesflorenciana* J. S. Moreno & Hoyos *	*Hoyos D., López O. & Fonseca A. 945* (COAH, HUAZ)	FLO
*Lepanthesforceps* Luer & R. Escobar	Living collection (El Caraño, Florencia)	FLO
*Lepantheshirtzii* Luer	*Trujillo E. 7687* (CUVC)	PRC
*Lepanthesmucronata* Lindl.	*Arias T. 1234* (HUAZ)	FLO
*Lepanthesnontecta* Luer **	iNaturalist	SVC
*Lepanthestachirensis* Foldats	*Arias T. 1232* (HUAZ)	FLO
*Lepantheswageneri* Rchb. f.	*Trujillo E. 7689* (CUVC)	PRC
*Lockhartiaacuta* Rchb.f.	iNaturalist	CAR
*Lockhartiamicrantha* Rchb. f.	*Betancur J. 2226* (COL, HUA), *Arias, T. 989* (HUAZ)	DON
*Lycastefuscina* Oakeley **	iNaturalist	SVC
*Lycastemacrobulbon* Lindl.	iNaturalist	SVC
*Lycastemacrophylla* Lindl.	iNaturalist	SVC
*Lycomormiumfiskei* H.R. Sweet	*Ortiz-Valdivieso M. 1365* (HPUJ)	N/A
*Lycomormiumschmidtii* Á. Fernández	*Fernandez 7248* (COAH)	FLO
*Macradeniapurpureorostrata* G. Gerlach	*Romero 4082* (COL)	PRC
*Macrocliniummanabinum* (Dodson C.) Dodson C. **	iNaturalist	FLO
*Malaxishistionantha* (Link, Klotzsch & Otto) Garay & Dunst.	iNaturalist	SVC
*Masdevalliaamanda* Rchb. f. & Warsz.	*Perdomo O. 395* (CUVC), *Ortiz-Valdivieso M. 4185* (HPUJ), *Arias T. 1187* (HUAZ)	FLO, PRC
*Masdevalliaconstricta* Poepp. & Endl. **	iNaturalist	FLO
*Masdevalliaensata* Rchb. f.	*Trujillo E. 7698* (CUVC)	FLO
*Masdevalliapicturata* Rchb.f.	*Arias T. 1230* (HUAZ)	FLO
*Masdevalliatubulosa* Lindl.	*Polania & Trujillo E. 10* (HUAZ), *Trujillo E. 7696* (CUVC)	FLO, PRC
*Masdevalliavirgo-cuencae* Luer & Andreetta	*Perdomo O. 423* (CUVC)	FLO, SVC
*Maxillariaacuminata* Lindl.	*Perdomo O.* 199 (CUVC)	FLO
*Maxillariaaequiloba* Schltr.	*Trujillo E. 7561, Perdomo O. 399* (CUVC)	FLO, PRC
*Maxillariaalba* Lindl.	*Ortiz-Valdivieso M. 467* (HPUJ)	BEL
*Maxillariaanceschiana* Molinari	*Correa M & Trujillo E. 5344* (HUAZ)	FLO
*Maxillariaaureoglobula* Christenson	*Perdomo O. 292, 349* (CUVC), *Chaux-Varela J. 36* (HUAZ)	BEL, FLO
*Maxillariaaurea* (Poepp. & Endl.) L. O. Williams	*Cardenas D. 48633* (COAH), *Sanin D. 6569* (COL), *Perdomo O. 411* (CUVC), *Diaz et al. 38, Santofimio L. M. & Trujillo E. 12, Molina A. 31, Arias T. 1202* (HUAZ)	FLO, PRC
*Maxillariaauyantepuiensis* Foldats	*Trujillo W. 1041* (COAH), *Ortiz-Valdivieso M. 556* (HPUJ)	BEL, FLO
*Maxillariabicallosa* (Rchb. f.) Garay	*Betancur J. 20637* (COAH), *Perdomo O. 370, 384* (CUVC), *Chaux-Varela J. 35* (HUAZ)	BEL, FLO
*Maxillariabolivarensis* C. Schweinf.	*Trujillo E. 957* (COAH), *Perdomo O. 346, 382* (CUVC)	BEL, FLO
*Maxillariabrachybulbon* Schltr.	*Perdomo O. 285* (CUVC)	FLO
*Maxillariabuchtienii* Schltr.	*Perdomo O. 316* (CUVC)	FLO
*Maxillariacamaridii* Rchb. f.	*Cardenas D. 41896, 44563* (COAH), *Trujillo E. 812* (HUAZ)	BEL, CAR, FLO, SVC
*Maxillariacarinulata* Rchb. f.	*Santofimio L. M. & Trujillo E. 6* (HUAZ)	FLO
*Maxillariacassapensis* Rchb. f.	*Perdomo O. 401, Trujillo E. 7700* (CUVC)	PRC
*Maxillariacrassifolia* (Lindl.) Rchb.f.	*Arevalo R. 68, 157, Franco-Rosselli P. 4153* (COL), *Perdomo O. 370* (CUVC)	BEL, SOL, PRC
*Maxillariacruentata* (Arévalo & Bergq.) Molinari & Mayta *	*Arevalo R. 1080* (COL, WIS)	SOL, SVC
*Maxillariacryptobulbon* Carnevali & J.T. Atwood	*Londoño 871* (UDBC)	PRC
*Maxillariacuzcoensis* C. Schweinf. **	*Dodson C. 3255* (SEL)	FLO
*Maxillariadiscolor* (G. Lodd. ex Lindl.) Rchb. f.	*Arevalo R.218, Betancur J. 20650, Pabon M. 544* (COAH), *Vasco A. 342* (HUA), *Arias T. 848, 849, 853, 867, Chaux-Varela J. 38, Trujillo E. 806* (HUAZ)	BEL, SOL, CAR, PRC, SVC
*Maxillariadunstervillei* Carnevali & I. Ramirez J. G. **	*Castaño N. 8572* (COAH)	BEL, PRC
*Maxillariaecuadorensis* Schltr.	*Perdomo O. 274* (CUVC), *Santofimio L. M. & Trujillo E. 10* (HUAZ)	FLO
*Maxillariaegertoniana* (Bateman) Molinari	Living collection (El Manantial, Florencia)	SJF
*Maxillariaembreei* Dodson C.	*Castaño N. 8843* (COAH), *Perdomo O. 321* (CUVC), *Araujo D. & Trujillo E. 3, Arias T 1219* (HUAZ)	BEL, FLO, SOL
*Maxillariaequitans* (Schltr.) Garay	iNaturalist	CAR
*Maxillariaerikae* Molinari **	*Perdomo O. 0167* (CUVC)	FLO
*Maxillariaexaltata* (Kraenzl.) C. Schweinf.	*Ortiz-Valdivieso M. 4172* (HPUJ), *Araujo D. & Trujillo E. 32* (HUAZ)	FLO
*Maxillariafractiflexa* Rchb. f.	*Perdomo O. 391* (CUVC)	PRC
*Maxillariaimbricata* Barb. Rodr.	*Arias, T. 957, 958, 970, 1016* (HUAZ)	DON, FLO, PAU, SVC
*Maxillariainaequisepala* (C. Schweinf.) Molinari	*Prado et al. 614* (FMB)	SOL
*Maxillariakegelii* Rchb.f.	*Arevalo R. 276* (COAH), *Correa M. 9932* (HUAZ)	SOL
*Maxillarialepidota* Lindl.	iNaturalist	FLO
*Maxillarialongipetala* Ruiz & Pav.	iNaturalist	SVC
*Maxillarialongipetiolata* Ames & C. Schweinf.	*Perdomo O. 262* (CUVC), *Trujillo E. 1041* (HUAZ)	BEL, FLO
*Maxillarialongissima* Lindl.	*Perdomo O. 233* (CUVC), *Araujo D. & Trujillo E. 4, Arias T. 1191* (HUAZ)	FLO
*Maxillariamapiriensis* (Kraenzl.) L. O. Williams	*Perdomo O. 323* (CUVC), *Arias T. 965* (HUAZ)	FLO, DON
*Maxillariameridensis* Lindl.	*Cuatrecasas J. 9121* (COL), *Araujo D. & Trujillo E. 4, Arias T.12078, Chaux-Varela J. 90, Cumaco L. S. & Trujillo E. 32, Pinilla J. et al. 32, Plazas L. L. et al. 37* (HUAZ)	FLO
*Maxillarianasuta* Rchb. f.	Living collection (El Manantial, Florencia)	SOL
*Maxillarianotylioglossa* Rchb. f.	*Perdomo O. 179* (CUVC)	FLO
*Maxillarianovoae* Molinari	*Perdomo O. 0173* (CUVC)	FLO
*Maxillarianubigena* (Rchb. f.) C. Schweinf.	*Correa M & Trujillo E. 4903, Santofimio L. M. & Trujillo E. 16* (HUAZ)	FLO
*Maxillariaobtusa* (Lindl.) Molinari	*Barbosa C. 7543* (COAH), *Franco-Rosselli P. et al. 3814* (COL, MO)	SOL
*Maxillariaparkeri* Hook.	*Gentry A. 65290* (COAH, MO) *Arevalo R. 154, 267, 325* (COL), *Prado L. F. 526, 542* (COAH, MO, COL)	SOL
*Maxillariaparviflora* (Poepp. & Endl.) Garay	*Gentry A. 65290* (COAH, MO) *Arevalo R. 154, 267, 325* (COL) *Arias T. 1001, Chaux-Varela J. 104* (HUAZ)	BEL, DON, FLO, SVC
*Maxillariapendens* Pabst **	Living collection (El Caraño, Florencia)	FLO
*Maxillariapergracilis* (Schltr.) Schuit. & M.W. Chase	*Perdomo O. 372* (CUVC)	BEL, FLO
*Maxillariaplicata* Schltr.	*Arias, T. 1205* (HUAZ)	FLO
*Maxillariaporrecta* Lindl.	*Perdomo O. 273, 340* (CUVC), *Arias T. 928, 963, 1204, Polania D. & Trujillo E. 6* (HUAZ)	BEL, DON, FLO
*Maxillariaproboscidea* Rchb. f. **	*Arias T. 850, 868* (HUAZ)	SOL
*Maxillariapterocarpa* Barb. Rodr.	*Dodson C. 3247* (SEL)	FLO
*Maxillariaringens* Rchb. f.	*Ortiz-Valdivieso M 529* (HPUJ)	FLO
*Maxillariasanantonioensis* Christenson *	Living collection (El Caraño, Florencia)	FLO
*Maxillariasetigera* Lindl.	*Cardenas D. 48631* (COAH)	FLO
*Maxillariasoulangeana* Molinari	Living collection (El Caraño, Florencia)	BEL
*Maxillariasplendens* Poepp. & Endl.	*Ortiz-Valdivieso M. 554, 4241* (HPUJ)	FLO
*Maxillariastriata* Rolfe	*Trujillo E. s.n.* (CUVC)	FLO
*Maxillariasubrepens* (Rolfe) Schuit. & M. W. Chase	*Arevalo R. 87* (COL)	SOL
*Maxillariatenuis* C. Schweinf.	*Arevalo R. 85, 362* (COAH, COL)	BEL, SOL
*Maxillariauncata* Lindl.	*Arevalo R. 213* (COAH), *Arias, T. 915* (HUAZ)	SOL
*Maxillariavillosa* (Barb. Rodr.) Cogn.	*Prado 508, Restrepo 866* (COAH)	SOL
*Maxillariaviolaceopunctata* Rchb. f.	*Sastre R. D. 5061* (P)	SOL
*Miltoniopsisphalaenopsis* (Linden & Rchb. f.) Garay & Dunst.	*Cabezas 1752* (JBB)	SVC
*Muscarellacryptophyta* (Barb.Rodr.) Bogarín & Karremans **	*Arias T. 918* (HUAZ)	FLO
*Muscarellasamacensis* (Ames) Luer	*Ortiz-Valdivieso M. 474* (HPUJ), *Chaux-Varela J. 53* (HUAZ)	FLO
*Myoxanthusaffinis* (Lindl.) Luer	Living collection (El Manantial, Florencia)	SJF
*Myoxanthuscimex* (Luer & R. Escobar) Luer	*Perdomo O. 266, 415* (CUVC)	FLO
*Myoxanthusmerae* (Luer) Luer **	*Arias T. 1020* (HUAZ)	SVC
*Myoxanthusmonophyllus* Poepp. & Endl.	*iNaturalist*	SVC
*Myoxanthusreymondii* (H. Karst.) Luer	*Arias T. 974, Chaux-Varela J. 86* (HUAZ)	DON
*Myoxanthusxiphion* Luer	*Perdomo O. 180, 414* (CUVC)	FLO
*Notyliabarkeri* Lindl. **	*Arias T. 846, 847* (HUAZ)	SOL
*Notyliaplatyglossa* Schltr.	*Perdomo O. 271* (CUVC)	VAL
*Notyliasagittifera* (Kunth) Link, Klotzsch & Otto	iNaturalist	MIL
*Octomeriacolombiana* Schltr.	*Trujillo E. 1039* (COAH, HUAZ), *Arias T.973, Chaux-Varela J. 87* (HUAZ)	DON, BEL
*Octomeriaerosilabia* C. Schweinf.	*Arevalo R 84* (COL), *Arevalo R. 242, van der Wal 231 M.* (COAH), *Vasco A. 242, 255* (HUA)	SOL
*Octomeriaexigua* C. Schweinf.	*Arevalo R. 356* (COL), *Gonzalez M. F. 2693* (COAH, COL)	FLO, SOL
*Octomeriagrandiflora* Lindl.	*Arevalo R. 367* (COL), *Mesa N. & Trujillo E. 07* (HUAZ)	FLO, SOL
*Octomeriaminor* C. Schweinf.	*Vasco A. 188, 203* (COL)	SOL
*Octomeriascirpoidea* (Poepp. & Endl.) Rchb.f.	*Cardenas D. 6854* (COAH), *Arevalo R. 273* (COL), *Vasco A. 202* (HUA)	SOL
*Octomeriasurinamensis* H. Focke	*Arevalo R. 90, 152, 246, 266, 348* (COL)	SOL
*Octomeriataracuana* Schltr.	*Velayos 6421* (MA), *Franco-Rosselli P. 4148* (COL)	SOL
*Octomeriatridentata* Lindl.	*Dodson C. 3245* (SEL)	FLO
*Odontoglossumpaniculatum* Dalström & Deburghgr.	iNaturalist	SVC
*Oliverianabrevilabia* (C. Schweinf.) Dressler & N.H. Williams	iNaturalist	SVC
*Oncidiumabortivum* Rchb. f.	*Betancur J. 2197* (HUA)	SVC
*Oncidiumalexandrae* (Bateman) M. W. Chase & N.H. Williams	*Luteyn J. L. et al. 4958*, *Sanin D. 6395* (COL), *Calderon A. 248* (HUAZ), *Gentry A. el at. 9183* (MO)	FLO, PRC
*Oncidiumbaueri* Lindl.	*Trujillo E. 549* (COAH, HUAZ), *Calderon A. 249 Arias T. 995, 1214* (HUAZ)	DON, FLO, SOL
*Oncidiumcitrinum* Lindl.	*Ortiz-Valdivieso M 550* (HPUJ)	FLO
*Oncidiumeliae* (Rolfe) M. W. Chase & N. H. Williams	*Perdomo O. 409* (CUVC)	FLO, PRC
*Oncidiumensatum* Lindl. **	iNaturalist	SVC
*Oncidiumfuscatum* Rchb. f.	*Correa M. et al. 5113* (HUAZ)	FLO
*Oncidiumgramineum* (Poepp. & Endl.) M. W. Chase & N. H. Williams	*Perdomo O. 362, 3623* (CUVC), *Arias T. 920, Chaux-Varela J. 52* (HUAZ)	BEL, FLO
*Oncidiumorthotis* Rchb. f.	*Perdomo O.* 348, 405 (CUVC)	BEL, PRC
*Oncidiumpoikilostalix* (Kraenzl.) M. W. Chase & N. H. Williams	iNaturalist	SVC
*Oncidiumputumayense* (P. Ortiz) *M. W. Chase & N. H. Williams*	iNaturalist	SVC
*Oncidiumsphacelatum* Lindl.	*Betancur J. 1666* (HUA)	SVC
*Ornithocephalus bryostachys Schltr.* **	*Hoyos F. s.n.* (HUAZ)	FLO
*Otoglossumglobuliferum* (Kunth) N. H. Williams & M. W. Chase	*Cardenas D. 48646* (COAH)	FLO
*Otoglossumserpens* (Lindl.) N. H. Williams & M. W. Chase	*Ramirez J. G. 5204* (COAH), *Perdomo O. 249, Trujillo E. 7623* (CUVC)	FLO
*Palmorchisguianensis* (Schltr.) C. Scweinf. & Correll	*Duivenvoorden J. 949* (MO, COAH)	SOL
*Palmorchispuber* (Cogn.) Garay	*Cardenas D. 48406, Castro F. 11280* (COAH)	SOL, SVC
*Paphiniacristata* (Lindl.) Lindl.	*Trujillo E. 3858* (HUAZ)	SOL
*Paphinialindeniana* Rchb. f.	*Bernal R. 533* (COL)	SJF
*Peristeriaguttata* Knowles & Westc.	*Sanchez M. 28* (HUAZ)	CAR
*Platystelealucitae* Luer	*Sanin D.* 6490 (COAH)	FLO
*Pleurothallisbivalvis* Lindl.	*Arias, T. 972* (HUAZ)	DON, FLO
*Pleurothallisbicornis* Lindl.	*Arias T. 1229* (HUAZ)	FLO
*Pleurothallischloroleuca* Lindl.	iNaturalist	SVC
*Pleurothalliscordata* (Ruiz & Pav.) Lindl.	*Perdomo O.* 422, 398 (CUVC), *Arias T. 1221* (HUAZ)	FLO, PRC
*Pleurothallisdiscoidea* Lindl.	*Arias, T. 953* (HUAZ)	PAU
*Pleurothallislanguida* Luer & R. Escobar	iNaturalist	SVC
*Pleurothallismanicosa* Luer & R. Escobar	iNaturalist	BEL
*Pleurothallismatudana* C. Schweinf.	Living collection (El Manantial, Florencia)	SJF
*Pleurothallismicrocardia* Rchb.f.	*Betancur J. 20415* (COAH)	BEL, SVC
*Pleurothallisoctavioi* Luer & R. Escobar	*Arias T. 1192* (HUAZ)	FLO, SVC
*Pleurothallisphalangifera* (C. Presl) Rchb. f.	*Trujillo E. 7946* (CUVC)	FLO
*Pleurothallispruinosa* Lindl	iNaturalist	BEL
*Pleurothallisruberrima* Lindl.	*Jimenez E. 33* (COAH)	FLO
*Pleurothallisruscifolia* (Jacq.) R. Br. in W. T. Aiton	*Castaño N. 9230* (COAH)	BEL, FLO
*Pleurothallissandemanii* Luer	Living collection (El Caraño, Florencia)	FLO
*Pleurothallissphaerantha* Luer **	Living collection (El Manantial, Florencia)	FLO
*Polycycnisbarbata* (Lindl.) Rchb. f.	*Perdomo O. 354, 378* (CUVC)	BEL, FLO
*Polyotidiumhuebneri* (Mansf.) Garay	*Benavides A. 1292* (HUA)	SOL
*Polystachyafoliosa* (Hook.) Rchb.f. in Walp.	*Cardenas D. 24841, Rodriguez W. D. 6973, Sanin D. 6465* (COAH), *Arias T. 855, 877, 1002, Trujillo E. & Marin 183, Cumaco L. S. & Trujillo E. 23, Trujillo E. et al. 1512* (HUAZ)	CAR, FLO, SOL, SVC
*Polystachyastenophylla* Schltr.	*Trujillo E. et al. 824, Mesa N. & Trujillo E. 8* (HUAZ)	CAR, MON
*Ponthievafertilis* (F. Lehm. & Kraenzl.) Salazar	iNaturalist	SVC
*Prescottiacordifolia* Rchb.f.	*Diaz J. 369* (COAH)	PRC
*Prescottiastachyodes* (Sw.) Lindl.	*Cuatrecasas J. 9070* (COL)	FLO
*Prosthecheaaemula* (Lindl.) W. E. Higgins	*Romero R. 4136* (COL, MO)	CAR
*Prosthecheachimborazoensis* (Schltr.) W. E. Higgins	*Mendoza H. 497* (FMB)	SOL
*Prosthecheacochleata* (L.) W. E. Higgins	iNaturalist	SVC
*Prosthecheacrassilabia* (Poepp. & Endl.) Carnevali & I. Ramírez	*Stevenson P. 961* (COAH), *Arias T. 994* (HUAZ)	FL, PAU, SVC
*Prosthecheagrammatoglossa* (Rchb.f.) W. E. Higgins	*Arias, T. 1018* (HUAZ)	SVC
*Prosthecheapygmaea* (Hook.) W. E. Higgins	iNaturalist	SVC
*Prosthecheatigrina* (Linden ex Lindl.) W. E. Higgins	Living collection (El Caraño, Florencia)	FLO, SVC
*Prosthecheavenezuelana* (Schltr.) W. E. Higgins	Living collection (El Manantial, Florencia)	N/A
*Prosthecheavespa* (Vell.) W. E. Higgins	*Arevalo R. 198, Castaño N. 8544, Cardenas D. 48640 (COAH), Perdomo O. 253, 367* (CUVC), *Santofimio L. M. & Trujillo E. 8, 15* (HUAZ)	BEL, FLO, SOL
*Psilochilusmacrophyllus* (Lindl.) Ames	*Betancur J. 20322, Castaño N. 7500, 8785* (COAH)	BEL
*Psychopsissanderae* (Rolfe) Lückel & Braem	Living collection (El Manantial, Florencia)	NA
*Pterostemmaescobarii* (Dodson C.) M. W. Chase & N. H. Williams	iNaturalist	SVC
*Rodrigueziabracteata* (Vell.) Hoehne	Living collection (El Manantial, Florencia)	ALB, BEL, FLO, SJF
*Rodrigueziaclaudiae* Chiron **	iNaturalist	SJF
*Rodrigueziachasei* Dodson & D. E. Benn.	iNaturalist	SVC
*Rodriguezialanceolata* Ruiz & Pav.	*Diaz et al. 101* (UDBC)	FLO, SOL
*Rudolfiellafloribunda* (Schltr.) Hoehne	*Ortiz-Valdivieso M. 999* (HPUJ)	PRC
*Rudolfiellapicta* (Schltr.) Hoehne	*Perdomo O. 280, 343* (CUVC)	BEL, FLO
*Sacoilalanceolata* (Aubl.) Garay	*Castro F. 67608* (COAH)	PRC
*Sarcoglottisneillii* Salazar & Tobar **	*Calderon A. 264* (HUAZ)	FLO
*Scaphosepalumcimex* Luer & Hirtz **	Living collection (El Caraño, Florencia)	FLO
*Scaphyglottisaurea* (Rchb.f.) Foldats	*Velayos 6870* (COL)	SOL
*Scaphyglottisbidentata* (Lindl.) Dressler	*Giraldo 3311* (COAH), *Estrada 666, Velayos 6514* (COL)	BEL, SJF
*Scaphyglottisboliviensis* (Rolfe) B. R. Adams	*Perdomo O. 428* (CUVC), *Arias T. 871, 903* (HUAZ)	BEL, FLO, CAR
*Scaphyglottiscaquetana* Szlach. & Kolan. *	*Cardenas D. et al. 6899* (COAH)	SJF
*Scaphyglottisgraminifolia* (Ruiz & Pav.) Poepp. & Endl.	*Perdomo O. 418* (CUVC), *Arias T. 905, Chaux-Varela J. 37* (HUAZ)	FLO, SJF, SOL
*Scaphyglottisimbricata* (Lindl.) Dressler	Living collection (El Manantial, Florencia)	SJF
*Scaphyglottislongicaulis* S. Watson	*Cumaco L. S. & Trujillo E. 7, Mesa N. & Trujillo E. 2, Santofimio & Trujillo E. 11* (HUAZ)	BEL, SJF
*Scaphyglottisobtusisepala* Szlach. & Kolan. *	*Trujillo E. 1042* (COAH)	BEL
*Scaphyglottisprolifera* (Sw.) Cogn.	*Aguilar M. 251* (COAH)	FLO
*Scaphyglottispunctulata* (Rchb. f.) C. Schweinf.	*Trujillo E. 7664, 7863* (CUVC), *Arias T. 96*6, *1200, 1209* (HUAZ)	DON, FLO, PRC
*Scaphyglottisstellata* Lodd. ex Lindl.	*Cardenas D. 48550, 6932, Gonzalez M. F. 2617, Rodriguez M. 3641, Stevenson P. 1399, Velayos M. 6412* (COAH), *Arevalo R. 88* (COL), *Trujillo E. 885* (HUA), *Arias T. 869, 905, 962, Chaux-Varela J. 42* (HUAZ), *Cardenas D. 6932* (MO)	CAR, DON, FLO, SJF, SOL, SVC
*Sertiferapurpurea* Lindl. & Rchb.f.	*Sanin D. 6098* (COAH)	FLO
*Sobraliabiflora* Ruiz & Pav.	*Trujillo E. 887* (HUAZ)	SJF
*Sobraliacrocea* (Poepp. & Endl.) Rchb. f.	*Cardenas D. 42426* (FBM, COAH), *Betancur J. 20496* (COAH), *Trujillo E. 7985* (CUVC)	BEL, FLO
*Sobraliadecora* Bateman	*Arevalo R. 346* (COAH)	SOL
*Sobraliafimbriata* Poepp. & Endl.	*Perdomo O. 337* (CUVC)	BEL
*Sobraliafragrans* Lindl.	*Palacios P. 2578* (COL), *Castaño N. 7582, Cardenas D. 46381, Betancur J. 20594* (FMB)	BEL, SOL
*Sobraliagranitica* G. A. Romero-Gonzalez MF & Carnevali	*Castro F. 10841* (COAH), *Arias T. 1015* (HUAZ)	SOL, SVC
*Sobraliaklotzscheana* Rchb. f.	*Betancur J. 20458* (COAH), *Romero R. 4053* (COL), *Arias T. 991, 1015* (HUAZ)	BEL, PAU
*Sobralialeucoxantha* Rchb. f.	*Polania D. & Trujillo E.* 4 (HUAZ)	FLO
*Sobralialiliastrum* Lindl.	*Arbelaez E. 326, Cardenas D. 4135, Palacios P. 1164* (COAH), *Franco-Rosselli P. 3237, 2417, 3718* (COL), *Cumaco L. S. & Trujillo E. 33, Pabon M. 971* (HUAZ), *Cardiel J. M.* 1010 (MA)	FLO, SOL
*Sobraliamacrophylla* Rchb. f	*Cuatrecasas J. 9119, Fernandez A. 20079* (COL), *Vasco A. 306* (HUA), *Mesa N. & Trujillo E.* 06, 22 (HUAZ)	FLO, SOL
*Sobraliaroezlii* Rchb. f.	iNaturalist	FLO
*Sobraliasessilis* Lindl.	Living collection (El Manantial, Florencia)	BEL
*Sobraliaviolacea* Linden ex Lindl	*Barbosa C. 8109, Gonzalez M. F. 2270, Palacios P. 2932, 2880* (COL)	SOL
*Sobraliavirginalis* Peeters & Cogn. In Cogn. & Goos.	iNaturalist	FLO, SVC
*Speckliniagrobyi* (Lindl.) F. Barros	*Escobar R. 5051* (MO)	SVC
*Speckliniapicta* (Lindl.) Pridgeon & M. W. Chase	*Cardenas D. et al. 48247* (COAH), *Arevalo R. 217* (COL)	SOL, SVC
*Stanhopeacandida* Barb. Rodr.	*Arias T. 854* (HUAZ)	SOL
*Stelisaviceps* Lindl.	*Cardenas D. et al. 41680* (COAH), *41773* (FMB)	BEL
*Steliskefersteiniana* (Rchb.f.) Pridgeon & M. W. Chase	iNaturalist	SOL
*Stelislindenii* Lindl.	*Ortiz-Valdivieso M. 536* (HPUJ)	BEL
*Stelisoblonga* (Ruiz & Pav.) Willd.	*Sanin D. 6492* (COAH, HUA)	FLO
*Stelispurpurea* (Ruiz & Pav.) Willd.	*Perdomo O. 268, 315* (CUVC)	FLO, SVC
*Stelissuperbiens* Lindl.	*Perdomo O. 368* (CUVC)	BEL
*Steniapallida* Lindl.	*Arias, T.* 913, 987 (HUAZ)	SJF, DON
*Telipogonpogonostalix* Rchb. f.	*Arias, T. 981* (HUAZ)	DON
*Telipogonpolymerus* Rchb. f. *	*Trujillo E. 7636, Perdomo O. 403* (CUVC)	FLO, PRC
*Telipogonselbyanus* N. H. Williams & Dressler **	*Perdomo O. 418* (CUVC)	FLO
*Trichocentrumcebolleta* (Jacq.) M. W. Chase & N. H. Williams	*Arias T. 909, 1000, 1010, Chaux-Varela J. 40* (HUAZ)	DON, FLO, SVC
*Trichocentrumhelicanthum* (Kraenzl.) J. M. H. Shaw	Living collection (El Manantial, Florencia)	N/A
*Trichocentrumnanum* (Lindl.) M.W. Chase & N. H. William	Living collection (El Manantial, Florencia)	SVC
*Trichocentrumnudum* (Bateman ex Lindl.) M. W. Chase & N. H. Williams	Living collection (El Manantial, Florencia)	FLO
*Trichocentrumpulchrum* Poepp. & Endl.	*Perdomo O. 276* (CUVC)	FLO
*Trichosalpinxorbicularis* (Lindl.) Luer	*Franco-Rosselli P. 4179, Palacios P. 2450, Arevalo R. 341* (COL), *Cardenas D. 42195* (FMB)	BEL, PRC, SOL
*Trizeuxis falcata Lindl.*	iNaturalist	BEL
*Tubellamulticuspidata* (Rchb.f.) Archila	*Cardenas D. et al. 41704* (COAH)	BEL
*Tubellapusilla* (Kunth) Archila	*Arias T. 870* (HUAZ), *Perdomo O. 397* (CUVC)	CAR, PRC
*Vanillabicolor* Lindl.	*Idobro J. M. 11423* (COAH)	SOL
*Vanillaguianensis* Splitg.	*Barona A. 4863 5192* (COAH)	CAR, SOL
*Vanillaodorata* C. Presl.	*Barona A. 4864* (COAH)	SOL
*Vanillapalmarum* (Salzm. ex Lindl.) Lindl.	*Barona 4620, Cardenas D. et al. 48604* (COAH)	BEL, SVC
*Vanillapenicillata* Garay & Dunst. in Dunst. & Garay	*Franco-Rosselli P. 4258* (COL)	SOL
*Vanillapompona* Schiede	*Cardiel J. M. 1089* (COL)	SOL
*Vanillasprucei* Rolfe	*Barona A. 3124, Duivenvoorden J. 719, Restrepo D. 441* (COAH)	SOL
*Vanillatrigonocarpa* Hoehne	*Barona A. 3125, 4611, 4612, 4613, 4615, 4616, 4617, 4618, 4619* (COAH)	BEL
*Warczewiczellaamazonica* Rchb. f. & Warsz.	*Alzate F. 980* (FAUC)	MIL
*Wullschlaegeliacalcarata* Benth.	*Cardenas D. et al. 48513* (COAH), *Blanco M. et al. 233* (COAH, HUAZ)	FLO
*Xerorchisamazonica* Schltr.	*Barona A. 1483* (COAH)	SOL
*Xerorchistrichorhiza* (Kraenzl.) Garay	*Franco-Rosselli P. 4240* (COL)	SOL
*Xylobiumfoveatum* (Lindl.) G. Nicholson	*Perdomo O. 324* (CUVC)	FLO
*Xylobiumleontoglossum* (Rchb. f.) Rolfe	*Trujillo E. et al. 7867* (CUVC)	FLO

### ﻿Field expeditions

To expand the checklist of Orchidaceae that occur in Caquetá, we carried out a total of 12 field expeditions between 2019 and 2023 for the project “Orquídeas para la Paz.” Two expeditions explored part of the Alto Fragua Indi Wasi National Natural Park in collaboration with the Park and ten expeditions explored montane, premontane forests and lowland Amazonian Forest of the Department. Fertile specimens were collected and prepared for herbaria according to techniques used for orchid collections, that includes the preservation of flowers in spirit collections, photographs and tissue collections for ongoing DNA analyses. The specimens were deposited at either the Universidad de la Amazonia (HUAZ) or the Universidad del Valle (CUCV) Herbaria (acronyms according to Thiers 2020). Duplicate collections were made for other herbaria when possible. Living specimens, collected when flowers were not found, were taken to local nurseries at El Caraño, Florencia, located at 950 m a.s.l. for cold weather orchids, or El Manantial, Florencia, located at 300 m a.s.l. for warm weather orchids. Once they flowered, they were photographed, identified and herbarium specimens were made. All collections were deposited under the collection permit of the Universidad de la Amazonia (permit number 01691 October 2020; Indi Wasi National Park memorandum No. 20182200004943) by Alexis Calderón, Marco Correa and Edwin Trujillo.

### ﻿Resources used

Databases and herbaria were used to find herbarium specimens that were examined to back-up observations without vouchers. Available literature, as well as local, regional and national catalogues were used to find herbarium specimens collected in the region. Only records with a herbarium specimen were considered for this checklist; living specimens and iNaturalist records by one of the authors or collaborators and with a specialist identification were considered in the list, but only as “tentative” until the herbarium collection is available.

To carry out online consultations in herbaria, search criteria were considered using the keywords: “Caquetá”, “Orchidaceae”, “tropical humid forest”, “botanical expeditions”, “Amazon region” and “Caquetá River.” The “advanced search” option was used for most of the herbaria consulted, since it allows for a more direct search for information. International herbaria consulted, either in person or online, included: Harvard University Oak Ames Herbarium (AMES), Herbario del Instituto Chinoin (AMO), Berlin (B), Royal Botanic Gardens Kew Herbarium (KEW), University of California, Los Angeles Herbarium (LA), the Real Jardín Botánico de Madrid (MA), Naturalis Biodiversity Center (NL), Herbarium Utrech (U), New York Botanical Garden (NY), the Muséum national d´Histoire naturelle in Paris (P), Marie Selby Botanical Gardens (SEL), Tropicos (2002) of the Missouri Botanical Garden (MO), Gdansk University (UGDA), University of Florida Herbarium (USF), W-Reichenbach (Vienna) and University of Wisconsin Herbarium (WIS).

Colombian herbaria included: Instituto Amazónico de Investigaciones Científicas – SINCHI (COAH), Herbario Nacional Colombiano (COL), Herbario de la Universidad del Cauca (CAUP), Herbario de la Universidad del Valle (CUCV), Herbario de la Universidad de Caldas (FAUC), Herbario Federico Medem-Bogotá (FMB), Herbario de la Pontificia Universidad Javeriana (HPUJ), Herbario de la Universidad de Antioquia (HUA), Herbario Enrique Forero (HUAZ) de la Universidad de la Amazonia, Jardín Botánico José Celestino Mutis de Bogota (JBB), Universidad de los Llanos (LLANOS), Universidad de Nariño (PSO), Universidad Surcolombiana (SURCO), Herbario Forestal de la Universidad Distrital Francisco José de Caldas (UDBC) and Universidad Pedagógica y Tecnológica de Colombia (UPTC). Other checked databases included GBIF (Global Biodiversity Information Facility, 2022), IDigBio (Integrated Digitized Biocollections) and BRAHMS at Marie Selby Botanical Gardens (Software for Natural History management).

### ﻿Name validation and data curation

Correct scientific names of species were assigned, based on the World Flora Online (WFO 2022), except for the genus *Tubella* (Luer) Archila, which was accepted as valid following Bogarín et al. (2018). All names were supported by herbarium specimens, photographs uploaded on iNaturalist (www.inaturalist.com) or living collections in one of the local nurseries that supported their presence in Caquetá with reliable taxonomic determination.

All records obtained from herbaria, databases and literature were carefully curated regarding their scientific name, locality, collector and collection number. For localities, names of municipalities were verified and updated. To assign a name to duplicates with different identification, the provenance of each assigned name was investigated, paying particular attention to plants identified by experts, curators and recognised taxonomists, besides using the date of determination and its citation in a publication. Lastly, using photographs and a list of taxonomic groups within orchids, we reached out to as many specialists as possible for their species expertise (see Acknowledgements). This final dataset was used for analyses.

### ﻿Data analysis

Collection records were georeferenced as precisely as the information allowed, since in many records, the location is not clearly specified, a frequent situation in orchids, old collections and those made by amateurs. Due to the lack of standardised geographical coordinates, only the number of species in municipalities is reported for this study. Georeferenced records and species distribution maps were constructed for the 16 municipalities and are available upon request.

## ﻿Results

A total number of 228 fertile specimens were collected in the field (collections made by Arias T., Chaux-Varela J., Perdomo O., Trujillo E. and Correa M.), 692 herbarium specimens were reviewed in different herbaria, 100 photographs and two living collections accessed for specimen identifications. The most abundant species in the field were *Epidendrumnocturnum* Jacq., *E.lacustre* Lindl., *Maxillariadiscolor* (G. Lodd. ex Lindl.) Rchb. f. and *Scaphyglottisstellata* Lodd. ex Lindl. Some of the rarest ones only observed in the field once were *Cyrtochilumcaquetanum* P. Ortiz, L. E. Álvarez & A. J. Carrillo, *Masdevalliaensata* Rchb. f. and *Paphiniacristata* (Lindl.) Lindl. In “Orquídeas para la Paz” expeditions, 98 species (collections made by Arias T. and Chaux-Varela J.) representing 29 genera were collected. Living individuals that were not flowering in the field were brought to El Manantial (300 m a.s.l.) and El Caraño (950 m a.s.l.) both located in the Municipality of Florencia. Herbaria collections are actively being made once orchids start flowering. A total of 55 species are available at El Manantial and 60 at El Caraño.

**Figure 2.**
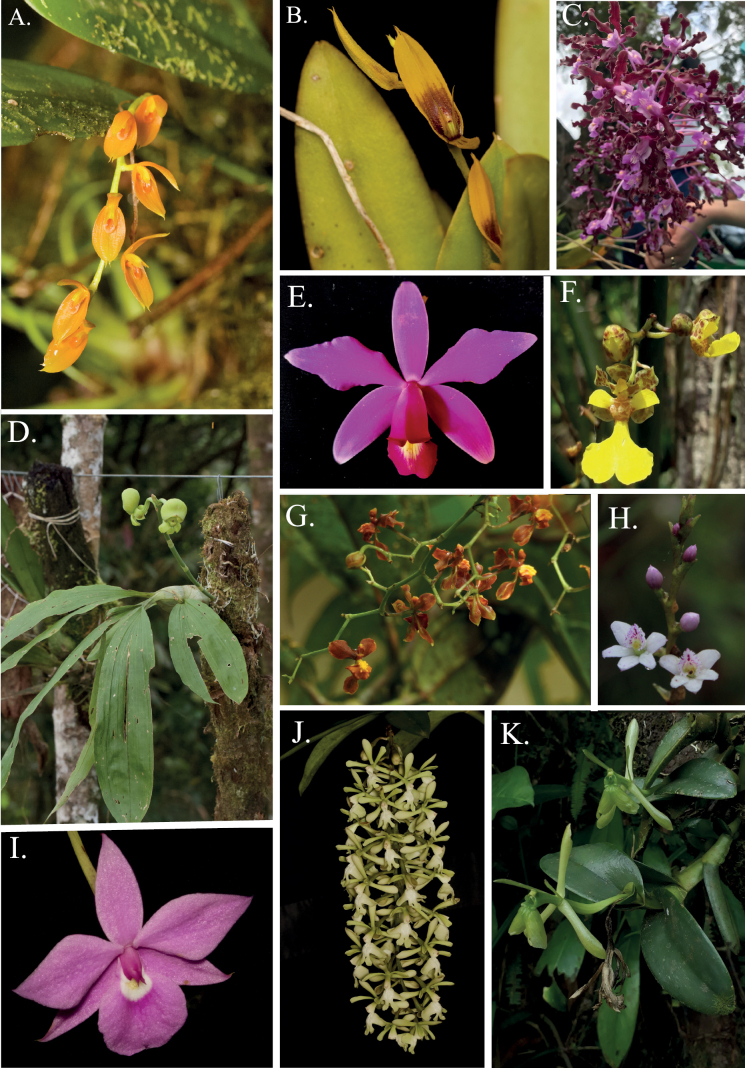
Representative Orchidaceae species from Caquetá, Colombia. **A***Aciantheracasapensis***B***Aciantheraciliata***C***Laeliarosea***D***Catasetumtuberculatum***E***Cattleyaviolacea***F***Trichocentrumnudum***G***Cyrtochilumporrigens***H***Epidendrumfimbriatum***I***Dimerandraemarginata***J***Epidendrumcoronatum***K***Epidendrumdifforme*.

**Figure 3.**
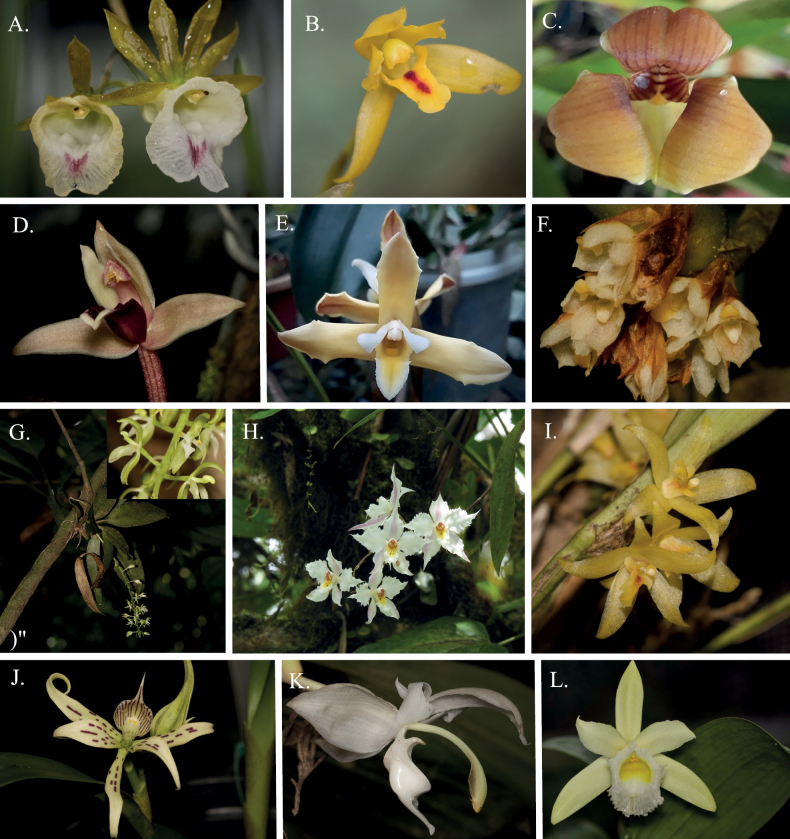
Representative Orchidaceae species from Caquetá, Colombia. **A***Galeandramacroplecta***B***Maxillariaaureoglobula***C***Maxillariaegertoniana***D***Maxillariaequitans***E***Maxillariaparkeri***F***Maxillariaparviflora***G***Notyliabarkeri***H***Oncidiumalexandrae***I***Octomeriagrandiflora***J***Prosthecheachimborazoensis***K***Stanhopeacandida***L***Sobraliamacrophylla*.

**Figure 4.**
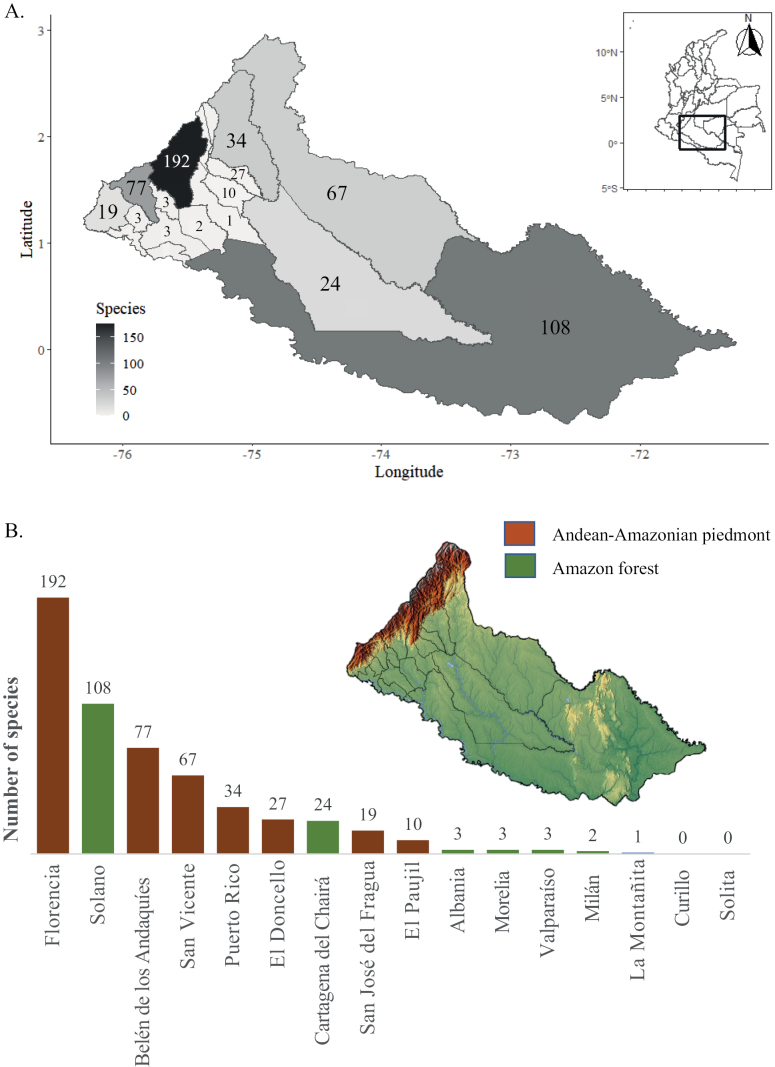
Distribution of orchid species number in Caquetá municipalities. **A** Orchid species distribution by municipality and a heatmap of species richness found in each of the municipalities **B** Number of orchid species found in each municipality and their geographic position (Andean Piedmont or Amazonian Basin).

**Figure 5.**
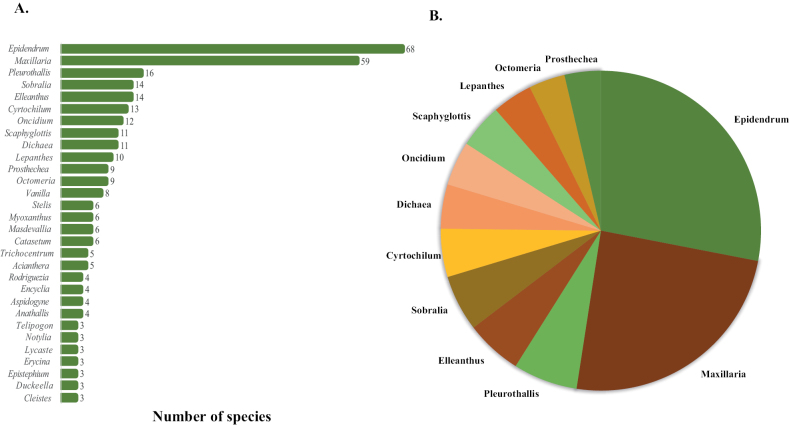
Number of orchid species in most abundant genera of Caquetá, Colombia **A** number of species in the most species rich genera of Caquetá **B** pie chart showing the proportion of species in genera with 9 to 70 species.

We report 418 species belonging to 98 genera represented in 744 herbarium collections including duplicates. Eighty-two species are new reports for Caquetá since they have not been vouchered until this study (Table 1, collections exclusively made by one of the authors of this paper). The most species-rich genera were *Epidendrum* L. (68 spp.), *Maxillaria* Ruiz & Pav. (59 spp.), *Pleurothallis* R.Br. (16 spp.), *Elleanthus* C. Presl (14 spp.), *Sobralia* Ruiz & Pav. (14 spp.) (Table 1, Figs 2–5). Most genera found in Caquetá (75) have one to three species (Fig. 5). We found two introduced species around urban areas of Florencia, *Arundinagraminifolia* (D. Don) Hochr. and *Dendrobiumnobile* Lindl.; these were not included in the species list.

Eighty-one species are included as “tentative” because they have been accurately identified, but lack a herbarium voucher. Fifty-three out of these 81 were included as potentially distributed in the Caquetá because records produced by the authors and collaborators in the field through photographs were not documented with herbarium vouchers. Such photographic records were submitted to iNaturalist (see https://www.inaturalist.org/projects/orquideas-del-caqueta). Twenty-seven species including 15 in El Manantial and 12 in El Caraño, are part of our living collections. They were collected fertile in the field and identified, but have not been photographed or documented with herbarium vouchers to date.

For the Municipality of Florencia, 192 species were recorded, followed by Solano with 108 and Belén de los Andaquíes with 77 (Fig. 4). Five municipalities including Albania, Morelia, Valparaíso, Milán and La Montañita have three or less records and there are no orchid botanical collections for Curillo and Solita (Table 1). Fourteen species are endemic to Colombia and from those, 11 have new herbarium vouchers made in this project. Twenty nine are new reports for Colombia and 15 of those have new herbarium vouchers as a result of this project (Table 1). *Masdevalliavirgo-cuencae* Luer & Andreetta (VU), *Miltoniopsisphalaenopsis* (Linden & Rchb. f.) Garay & Dunst. (VU) and *Oncidiumalexandrae* (Bateman) M. W. Chase & N. H. Williams (EN) were included in the Red List of Colombian orchid species (Calderón-Sáenz 2007).

Most collections made in Caquetá have been deposited at HUAZ, which currently holds 210 orchid specimens, while COAH holds 207 orchid specimens and COL 109 orchid specimens. Seven international herbaria had between 1–12 collections from Caquetá (Table 1).

## ﻿Discussion

A total of 276 new species records of Orchidaceae were added to the previous orchid report of Betancur et al. (2015), who cited 142 species. The great diversity of Orchidaceae species in Caquetá might be explained by spatial heterogeneity and phytophysiognomies in this region (Etter et al. 2006). The significant diversity of *Epidendrum* (68/1000) and *Maxillaria* (59/570) was expected because these are some of largest Neotropical Orchidaceae genera with regards to species number (Fig. 5, Suppl. material 1: table S1). The four most species-rich genera account for 40% of the total species, but they represent 4.12% of the total genera. Forty-three genera included only one species for the region, which corresponds to 19% of the total species and 42% of the total genera. Species in genera, such *Encyclia* Hook. and *Stelis* Sw., were challenging to identify and additional taxonomic work is required. One widely distributed and unpublished hybrid *Epidendrum×communis* Hágsater Ined. was added to the list after specialist advice (Hágsater, pers. comm.)

During the construction of this list, we left out collections made by Werner Hopp (Schlechter 1924) since they were collected in Putumayo in 1921 and 1922 when Putumayo was part of the Caquetá intendancy. In 1991, Putumayo was politically recognised as a Department and, as such, it is no longer part of Caquetá. Additionally, some species collected by Hopp were deposited in the Berlin Herbarium (B) and destroyed during Second World War (Suppl. material 1. table S2).

**Figure 6.**
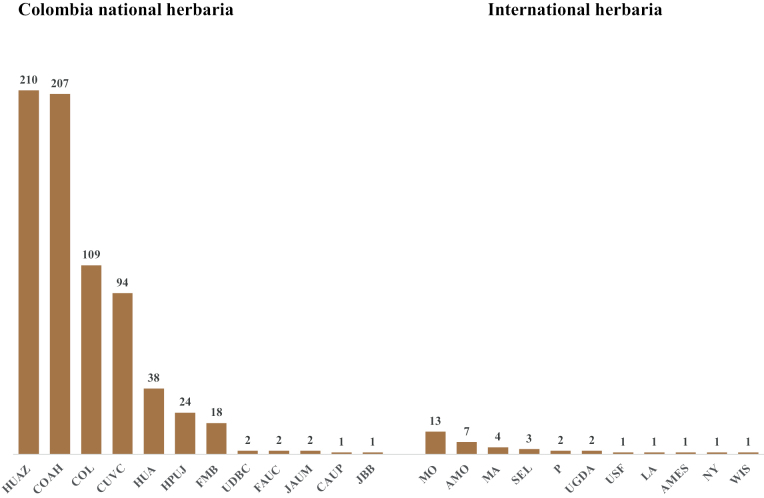
Number of orchid species in the Colombian national herbaria and international herbaria. Instituto Amazónico de Investigaciones Científicas – SINCHI (COAH), Herbario Nacional Colombiano (COL), Herbario de la Universidad del Cauca (CAUP), Herbario de la Universidad del Valle (CUCV), Herbario de la Universidad de Caldas (FAUC), Herbario Federico Medem-Bogotá (FMB), Herbario de la Pontificia Universidad Javeriana (HPUJ), Herbario de la Universidad de Antioquia (HUA), Herbario Enrique Forero (HUAZ) de la Universidad de la Amazonia, Jardín Botánico José Celestino Mutis de Bogotá (JBB), Universidad de los Llanos (LLANOS>), Universidad de Nariño (PSO), Universidad Surcolombiana (SURCO), Herbario Forestal de la Universidad Distrital Francisco José de Caldas (UDBC), and Universidad Pedagógica y Tecnológica de Colombia (UPTC).Harvard University Oak Ames Herbarium (AMES), Herbario del Instituto Chinoin (AMO), Berlin (B), Royal Botanic Gardens Kew Herbarium (KEW), University of California, Los Angeles Herbarium (LA), the Real Jardín Botánico de Madrid (MA), Naturalis Biodiversity Center (NL) - Botany (Herbarium Utrech), New York Botanical Garden (NY), the Herbier Museum Paris of the Museum National D´Histoire Naturelle (P), Marie Selby Botanical Gardens (SEL), TROPICOS database (access 2022) of the Missouri Botanical Garden (MO), Gdansk University (UGDA), University of Florida Herbarium (USF), W-Reichenbach (Vienna) and University of Wisconsin Herbarium (WIS).

Most orchid species documented in Caquetá are found in the Florencia Municipality (192 spp.). This could be explained by the convenience of collecting around cities and the wide altitudinal gradient in this municipality. We present collections numbers by municipality because conservation strategies might differ between political boundaries in Colombia. Regional Autonomous Corporations (CAR) are the main environmental authority. They are responsible for implementing policies and plans from the Ministry of Environment and are granted administrative and financial autonomy. Entities responsible for the formulation of such conservation strategies might benefit more from having such information presented following Departmental divisions. By depositing 140 herbarium specimens at HUAZ (duplicates will be sent to other herbaria) in the framework of this study, we substantially increased its orchid collection to 210 species, positioning HUAZ as the first local herbarium with more herbarium collections from Caquetá than any other herbaria (Fig. 6, Suppl. material 1: table S1).

Five of the municipalities of Caquetá, representing ~ 20% of the total geographical area of the Department, had zero to three herbarium collections or species reported (Fig. 4). The areas in the north-eastern part of the eastern Andean Mountain range still need extensive exploration. These areas include the National Natural Park Cordillera de Los Picachos, where landmines were planted by rebel groups during the long armed conflict that lasted for decades and these have not been removed to date. Orchid diversity could significantly increase with the development of intensive exploration in these mountainous ecosystems and a thorough exploration of the Amazonian Forest canopy. For instance, Departments like Antioquia and Huila have been catalogued as having the largest orchid diversity (Betancur et al. 2015); however, these areas of Colombia have not been extensively explored for decades.

During our expeditions, two species *Cattleyaviolacea* (Kunth) Lindl. and *Trichocentrumlanceanum* (Lindl.) M. W. Chase & N. H. Williams have been found only in La Laguna del Chaira in the Cartagena del Chaira Municipality. We doubt these species have a natural distribution there. Rather, we suspect they were introduced during the massive effort to bring orchids to La Laguna del Chaira during the 1980s, during which “uninformed” reintroductions of non-native species could have taken place.

This checklist places Caquetá as the eighth Department in Colombia in terms of genera diversity (98 genera) from its original position in the National Plan of Orchid Conservation (15^th^ place, 62 genera). As for the ranking in species number for Colombia, Caquetá goes from position 17^th^ (142 spp.) to position 9^th^ (418 spp.) (Betancur et al. 2015). Caquetá has many orchid genera (98/258 in Colombia) with few species each, 76% of genera having around 1–3 species. Each of these genera include a unique clade distributed in a relatively small area of Colombia. This could be of particular interest in conservation, prioritising evolutionary history over species diversity (Arponen 2012). Caquetá would be one of the regions of Colombia where there are more different genera represented in clades than in other Colombian regions. This work supplies valuable evidence to promote conservation efforts and politics for habitat preservation of the Colombia Andean Piedmont.

Caquetá has lost approximately 30% of its original area due to human impacts, such as cattle ranching. National parks in Caquetá make up 65% of the protected remnants. In the last 50 years, expansion of the agricultural frontier for the establishment of grazing lands, wood extraction and illegal coca crops have destroyed many ecosystems, greatly impacting all national parks. Florencia, for example, is currently undergoing consistent expansion of farming lands ultimately leading to the decimation of natural ecosystems (IDEAM 2020).

## ﻿Conclusion

Our floristic study is a needed contribution towards a better understanding of the diversity of Colombian orchids. The checklist provides a set of freely-available data on orchid diversity in Caquetá. Furthermore, our study is a baseline panorama of orchid species diversity in the Department, identifying groups of interest for further taxonomic work, especially those which have not been monographed. Lastly, the information provided could enhance local conservation strategies for endangered floristic elements in the Department by adding to a more complete overview of the high orchid diversity in the region.

## ﻿Additional information

### Author ORCIDs

Tatiana Arias https://orcid.org/0000-0001-8261-0635

R. Alexis Calderón-Álvarez https://orcid.org/0000-0001-5277-3070

Marco A. Correa-Munera https://orcid.org/0000-0003-2683-7733

Alejandro Zuluaga https://orcid.org/0000-0002-5874-6353

Oscar Perdomo https://orcid.org/0000-0003-1260-1965

Oscar A. Pérez-Escobar https://orcid.org/0000-0001-9166-2410

Edwin Trujillo-Trujillo https://orcid.org/0000-0002-5601-540X

Janice Valencia-D. https://orcid.org/0000-0003-2508-9825

### Data availability

All of the data that support the findings of this study are available in the main text or Supplementary Information.

## Supplementary materials

Supplementary material 1 Supplementary data Data type tables (excel document) https://binary.pensoft.net/file/873833This dataset is made available under the Open Database License (http://opendatacommons.org/licenses/odbl/1.0/). The Open Database License (ODbL) is a license agreement intended to allow users to freely share, modify, and use this Dataset while maintaining this same freedom for others, provided that the original source and author(s) are credited.Tatiana Arias, Jeisson Chaux-Varela, Maria del Pilar Camero, R. Alexis Calderón-Álvarez, Anyi Carolina Trujillo, Marco A. Correa-Munera, Alejandro Zuluaga, Oscar Perdomo, Oscar A. Pérez-Escobar, Edwin Trujillo Trujillo, Janice Valencia-D.
